# *RAD51C* Germline Mutations in Breast and Ovarian Cancer Cases from High-Risk Families

**DOI:** 10.1371/journal.pone.0025632

**Published:** 2011-09-28

**Authors:** Jessica Clague, Greg Wilhoite, Aaron Adamson, Adam Bailis, Jeffrey N. Weitzel, Susan L. Neuhausen

**Affiliations:** 1 Division of Clinical Cancer Genetics, Beckman Research Institute at the City of Hope National Medical Center, Duarte, California, United States of America; 2 Department of Population Sciences, Beckman Research Institute at the City of Hope National Medical Center, Duarte, California, United States of America; 3 Department of Molecular and Cellular Biology, Beckman Research Institute at the City of Hope National Medical Center, Duarte, California, United States of America; Ohio State University Medical Center, United States of America

## Abstract

*BRCA1* and *BRCA2* are the most well-known breast cancer susceptibility genes. Additional genes involved in DNA repair have been identified as predisposing to breast cancer. One such gene, *RAD51C*, is essential for homologous recombination repair. Several likely pathogenic *RAD51C* mutations have been identified in *BRCA1*- and *BRCA2*-negative breast and ovarian cancer families. We performed complete sequencing of *RAD51C* in germline DNA of 286 female breast and/or ovarian cancer cases with a family history of breast and ovarian cancers, who had previously tested negative for mutations in *BRCA1* and *BRCA2*. We screened 133 breast cancer cases, 119 ovarian cancer cases, and 34 with both breast and ovarian cancers. Fifteen DNA sequence variants were identified; including four intronic, one 5′ UTR, one promoter, three synonymous, and six non-synonymous variants. None were truncating. The in-silico SIFT and Polyphen programs were used to predict possible pathogenicity of the six non-synonomous variants based on sequence conservation. G153D and T287A were predicted to be likely pathogenic. Two additional variants, A126T and R214C alter amino acids in important domains of the protein such that they could be pathogenic. Two-hybrid screening and immunoblot analyses were performed to assess the functionality of these four non-synonomous variants in yeast. The RAD51C-G153D protein displayed no detectable interaction with either XRCC3 or RAD51B, and RAD51C-R214C displayed significantly decreased interaction with both XRCC3 and RAD51B (p<0.001). Immunoblots of RAD51C-Gal4 activation domain fusion peptides showed protein levels of RAD51C-G153D and RAD51C-R214C that were 50% and 60% of the wild-type, respectively. Based on these data, the *RAD51C*-G153D variant is likely to be pathogenic, while the *RAD51C*- R214C variant is hypomorphic of uncertain pathogenicity. These results provide further support that *RAD51C* is a rare breast and ovarian cancer susceptibility gene.

## Introduction

Breast cancer is the most common cancer worldwide and the second leading cause of cancer death among women in the United States [1]. Familial cases of breast cancer comprise approximately 5–10% of all breast cancer, whereas familial ovarian cancer accounts for 10% of invasive ovarian cancer [2], [3]. *BRCA1* and *BRCA2* are the most well-known genes predisposing to breast cancer. Mutations in *CHEK2*, *ATM*, *BRIP1*, and *PALB2*, genes also involved in genome maintenance and homologous recombination, have been identified as predisposing to breast cancer; however they only account for a small portion of the hereditary cases [4].

Recently, *RAD51C*, essential for homologous recombination repair, has been reported to be a rare hereditary breast and ovarian cancer susceptibility gene and several pathogenic *RAD51C* mutations have been identified in *BRCA1*- and *BRCA2*-negative hereditary breast and ovarian cancer families (HBOC) [5]. At the same time, a biallelic mutation in *RAD51C* was reported in a family with multiple severe abnormalities characteristic of Fanconi Anemia [6]. Several pathogenic variants were observed in five studies [5], [7], [8], [9], [10] with all in HBOC families or in ovarian cancer cases. No clearly pathogenic mutations were detected in two other studies [11], [12]. Thus, there are multiple studies and evidence that *RAD51C* is a rare ovarian cancer predisposition gene important in both breast and ovarian cancers in HBOC families. More studies are needed to determine its penetrance and the role it plays in these cancers.

In the current study, we screened for *RAD51C* mutations in a clinic-based set of women with breast and/or ovarian cancers in families with HBOC who had previously tested negative for *BRCA1* and *BRCA2* mutations.

## Results

### Mutation screening

We found 15 variants, including four intronic, one 5′ untranslated region (UTR), one promoter, three synonymous, and six non-synonymous variants (Table 2). None of the variants caused protein truncation. Of the fifteen variants, six had been previously identified and had dbSNP rs numbers, and nine were novel.

### Functional Analyses

**Figure 1:**
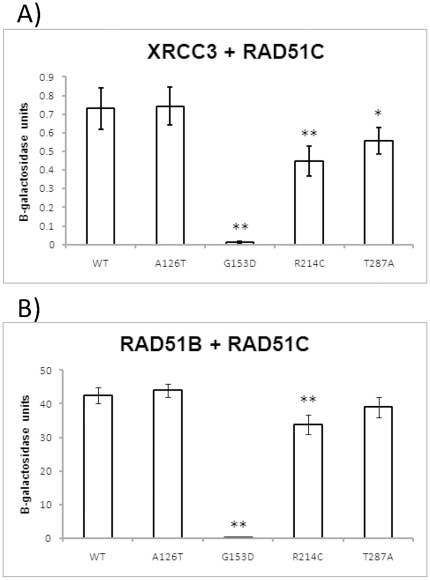
Effect of *RAD51C* point mutations on the interaction with XRCC3 and RAD51B. (A) Yeast two-hybrid assays were performed with XRCC3 in the DNA-binding domain vector and either wild-type or site-specifically mutated *RAD51C* in the activating domain vector. (B) Yeast two-hybrid assays were performed with RAD51B in the DNA-binding domain vector and the *RAD51C* variants in the activating domain vector. Results from liquid ONPG assays are the average of 5–7 different transformants performed in triplicate, with the standard error of the mean. * : *P*<0.05 and ** :*P*≤0.001 using the student *T*-test.

Of the six non-synonomous variants, SIFT and Polyphen predicted that two are likely pathogenic (G153D and T287A), based on the degree of conservation of the affected residue. An additional two non-synonomous variants (A126T and R214C) alter amino acids in conserved domains of the protein, and therefore could possibly disrupt function (Table 2). It had previously been shown through yeast two-hybrid analysis that human RAD51C interacts with human XRCC3 and RAD51B [13], [14], and that these proteins form complexes *in vivo* [14], [15], [16], [17]. We tested whether these missense mutations affected the interaction between RAD51C and both XRCC3 and RAD51B by yeast two-hybrid analysis. RAD51C-A126T displayed a level of interaction with both XRCC3 and RAD51B that was not significantly different from wild-type (p = 0.88 and 0.48, respectively), while interactions between RAD51C-G153D and these proteins were undetectable (p<0.0001 for both) (Figure 1). RAD51C- R214C displayed significant decreases in interaction with both XRCC3 (p = 0.0008) and RAD51B (p = 0.0002), while RAD51C-T287A displayed a slight but significant decrease in association with XRCC3 (P = 0.034 ), but a level of interaction with RAD51B that was not significantly different from wild-type (p = 0.11).

**Figure 2:**
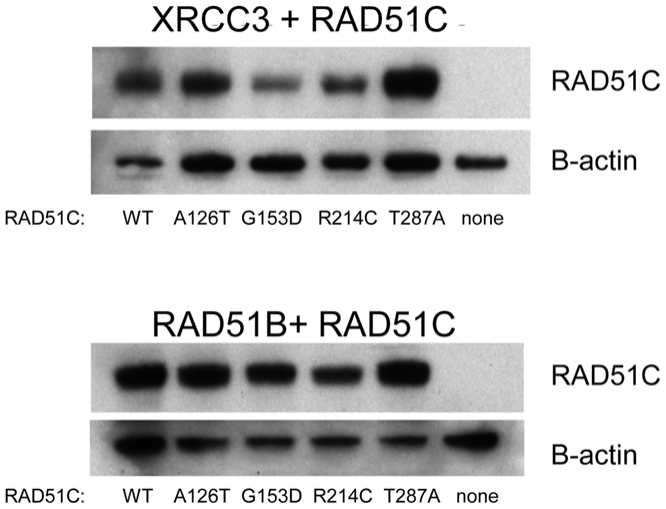
Immunoblots of *RAD51C* in the yeast strains used for the two-hybrid analysis . The *upper panel* shows the immunoblot blot analysis of the yeast strains containing human XRCC3 and RAD51C while the *lower panel* shows the same immunoblots from the yeast strains used in the RAD51B and RAD51C analysis. The RAD51C-Gal4 fusion peptides were identified using an anti-Gal4 monoclonal antibody. The same lysates were probed with an antibody against β-actin as a control for equal loading of protein.

One possible explanation for the decreased levels of interaction conferred by RAD51C-G153D, RAD51C-R214C, and RAD51C-T287A is that these mutations result in reduced steady-state levels of RAD51C. This could be caused by a reduction in gene expression or reduced protein stability due to improper folding. To rule out the possibility that significant differences in the steady-state levels of the mutant proteins in yeast could account for the observed differences in the two-hybrid assay results, we examined the steady-state levels of the wild-type and mutant RAD51C-Gal4 activation domain (AD) fusion peptides by western blot analysis using an antibody against the Gal4 AD (Figure 2). Blots were also probed with an antibody against β-actin as a control for equal loading of protein. As shown in Figure 2, the *RAD51C*-G153D mutation had little effect on RAD51C-AD in strains that were expressing XRCC3- and RAD51B-Gal4 DNA binding domain (BD) fusions respectively. The reduction (∼50%) in the strain also expressing XRCC3 was not sufficient to explain the complete absence of interaction with the protein indicated by the two-hybrid assay (Figure 1). Similarly, the *RAD51C*-R214C mutation had little effect on the RAD51C-AD in the strains that were also expressing XRCC3-BD and RAD51B-BD, respectively (Figure 2). Neither *RAD51C*-A126T nor *RAD51C*-T287A conferred detectable changes in the levels of RAD51C-AD. Therefore, there was no evidence for gross changes of the stability of RAD51C-AD in these yeast strains.

### Pedigree analysis

**Figure 3:**
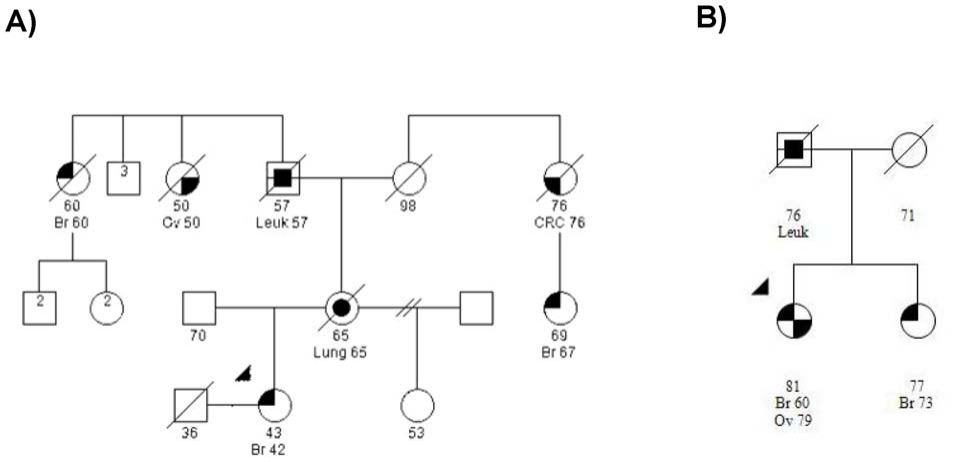
Pedigrees of cases carrying the R214C and G153D mutations. (A) R214C: African-American proband diagnosed with stage IIA, ER/PR positive, Her2/Neu positive, infiltrating ductal carcinoma of the breast at age 42 years. (B) G153D: Non-Hispanic Caucasian proband diagnosed with breast cancer at age 60 years and with stage IV, serous carcinoma of the ovary at age 79 years.

*RAD51C*-R214C is an exon 4 mutation and was found in an African-American proband who was diagnosed with stage IIA, ER/PR positive, Her2/Neu positive, infiltrating ductal carcinoma of the breast at age 42, and who had a family history of both breast and ovarian cancers on her mother's side of the family (Figure 3A). One great aunt on her grandfather's side was diagnosed with ovarian cancer at age 50 years and another great aunt with breast cancer was diagnosed at age 60 years. A first cousin once removed on her grandmother's side was diagnosed with breast cancer at age 67 years. We did not observe this variant in a set of 192 African-American women screened for mutations in *RAD51C* (data not presented). *RAD51C*-G153D is an exon 3 mutation identified in a Non-Hispanic White proband who was diagnosed with breast cancer at age 60 and with stage IV, serous carcinoma of the ovary at age 79. Her sister was diagnosed with breast cancer at age 73 (Figure 3B). No additional family DNAs were available to investigate co-segregation of the mutations with cancer.

## Discussion

It has been well established that deficient DNA repair and specifically, homologous recombination plays a critical role in cancer susceptibility. RAD51C is involved in two specific subcomplexes, one with XRCC2, RAD51B, and RAD51D, and the other with XRCC3 [18], and has multiple functions in the DNA damage response and the maintenance of genomic stability [19]. Interestingly, *RAD51C* is located in chromosomal region 17q23, which is amplified in primary breast cancer tumors [20], but it hadn't been investigated previously as a susceptibility locus for breast cancer. Further confirmation of its role in breast tumors was the discovery that the MCF-7 breast cancer cell line contains a *RAD51C-ATXN7* fusion gene consisting of *RAD51C* exons 1–7 and *ATXN7* exons 6–13 [21]. Other RAD51 gene family members had been associated with increased risk of breast cancer [18], but there had been no reports implicating *RAD51C*.

To further investigate the role of *RAD51C* as an HBOC predisposition gene, we performed complete sequencing of *RAD51C* to screen for mutations in 286 *BRCA1*- and *BRCA2*-negative breast and/or ovarian cancer cases with a family history of breast and ovarian cancer. Fifteen variants were identified, of which we selected four non-synonymous variants for functional studies; *RAD51C-G153D* and *RAD51C*-T287A because they alter highly conserved amino acid residues (predicted to be pathogenic by SIFT and Polyphen), *RAD51C*-A126T because it alters an amino acid in the Walker A box, and *RAD51C*-R214C because it alters an amino acid that separates a beta-strand from an alpha-helix. Yeast two-hybrid and immunoblot assays indicated that the *RAD51C*-T287A and *RAD51C*-A126T mutations do not substantially alter either the ability of RAD51C to interact with its companion proteins or its steady-state level, consistent with the results of the functional assays of Meindl at al. [7]. Furthermore, these two variants have been identified multiple times in other studies [5], [7], [8], [10], [11], [12], suggesting that they are benign polymorphisms. However, the results of the yeast two-hybrid and immunoblot assays were consistent with *RAD51C*-G153D being a pathogenic mutation as it led to undetectable levels of interaction between RAD51C and both XRCC3 and RAD51B, but did not markedly change levels of RAD51C. We hypothesize that the *RAD51C*-G153D mutation critically alters the ability of RAD51C to interact with XRCC3 and RAD51B, although it is not in a region of known secondary structure. *RAD51C*-R214C may be a hypomorphic mutation of uncertain clinical significance. Small but significant reductions in interaction between RAD51C and both XRCC3 and RAD51B were observed, but there were no marked changes in the steady-state level of RAD51C (Figures 1 and 2). Unfortunately, there were no additional samples in the families in order to investigate cosegregation with breast and ovarian cancer or tumor tissue to determine if there was loss of heterozygosity of the wild-type allele.

*RAD51C* first was identified as a rare hereditary breast and ovarian cancer (HBOC) predisposition gene by Meindl et al. [5]. After discovering that it was associated with Fanconi anemia [6], they screened for mutations in *RAD51C* in 1100 hereditary breast (HBC) and HBOC families, hypothesizing that it would be similar to *BRIP1* and *BRCA2* in which biallelic mutations cause Fanconi anemia and monoallelic mutations cause HBOC. They identified 14 monoallelic germline mutations of which 6 were considered pathogenic [5]. The mutations were only identified in cases from families with both breast and ovarian cancers (6/480) and in no cases from families with only breast cancer (0/620) [5]. In a Spanish study of 492 *BRCA1*- and *BRCA2* tested-negative breast cancer patients with family history of breast and/or ovarian cancer, they identified 12 variants, of which one was clearly pathogenic in the subset of 106 cases with a family history of both breast and ovarian cancers [7]. Interestingly, the one case was of Swedish origin, and this mutation was recently reported in an ovarian case in a study of Swedish and Finnish familial breast cancer cases and unselected ovarian cancer cases [8]. That study also reported a second clearly pathogenic mutation in an HBOC breast cancer case. In a recent Finnish study, two recurrent deleterious mutations were identified, and specifically in those with a personal or family history of ovarian cancer [10]. In a Chinese study of 275 women from HBOC families, two possibly pathogenic mutations were found [9]. In two additional studies of 454 and 92 breast and/or ovarian cancer cases from HBOC families, no pathogenic mutations were found [11], [12]. *RAD51C* mutations appear to be rare mutations that predispose to ovarian cancer, as well as to breast cancer but only in families with ovarian cancer.

In conclusion, we identified one likely pathogenic mutation and one hypomorphic mutation. These unique mutations had not been seen in previous studies, in this case series or in a case series of African Americans. The likely pathogenic mutation G513D mutation was found in a woman with both breast and ovarian cancers and the likely hypomorphic mutation, R214C mutation was found in an African-American woman with breast cancer and a family history of ovarian cancer. Our results provide further data that *RAD51C* is a predisposition gene for hereditary breast and ovarian cancers. Future studies investigating larger, multi-ethnic populations of ovarian cancers, and that include sampling of family members, are needed to better understand the role of *RAD51C* in ovarian cancers and HBOC.

## Methods

### Ethics Statement

All research involving human participants was approved by the City of Hope Institutional Review Board (IRB#96144). After a complete description of the study to the subjects, written, informed consent was obtained.

### Study Subjects

The study population was women diagnosed with breast and/or ovarian cancers seen for genetic cancer risk assessment (GCRA) in The City of Hope Clinical Cancer Genetics Community Research Network and enrolled in an Institutional Review Board-approved registry protocol (IRB#96144) between October 1996 and May 2010. The Clinical Cancer Genetics Community Research Network is a collaboration of genetic cancer risk assessment programs. All of the programs follow standard genetic cancer risk assessment protocols, including counseling by experienced clinicians, and assembly of a 4–5 generation pedigree with detailed cancer histories. A detailed family history of at least three generations was obtained at the initial GCRA visit and each patient donated a blood sample after written, informed consent.

Eligibility criteria for the current study included (1) a diagnosis of breast and/or ovarian cancer, (2) a previous negative test result for pathogenic germline mutations in *BRCA1* and *BRCA2*, and (3) a family history of at least one breast cancer and/or ovarian cancer in a first-, second-, or third-degree relative. A total of 133 breast cancer cases, 119 ovarian cancer cases, and 34 cases with both breast and ovarian cancer were eligible. All cases were unrelated. Clinical diagnosis and race/ethnicity information are described in Table 1.

**Table 1:** Characteristics of tested individual in family.

Cancer Diagnosis	Total	Non-HispanicWhiteN (%)	HispanicN (%)	OtherN (%)	MissingN (%)	Age of onset of first primary diagnosis in years(mean ± SD)
Breast and Ovarian	43	7 (20.6)	6 (17.7)	16 (18.6)	14 (41.2)	52.6±13.7
Breast	119	52 (43.7)	12 (10.1)	6 (5.1)	49 (41.2)	51.4±13.1
Ovarian	124	54 (40.6)	12 (9.0)	11 (17.1)	47 (35.3)	42.9±6.7
Total	286	113 (39.5)	30 (10.5)	33 (11.5)	110 (38.5)	47.6±11.5

**Table 2:** Variants identified through sequencing.

Variant	RS#	Location	Effect*	Amino Acid	Functional prediction	Observed MAF	Hapmap MAF	Previously Observed
c.1-118G>A	rs16943176	Promoter				0.155	0.203	[12]
c.1-26C>T	rs12946397	5′ UTR				0.166	0.207	[10], [12]
c.186A>G	rs28363303	Exon 2	S	Q62Q		0.002	0.006	[12]
c.336G>C	–	Exon 2	S	G112G		0.002		
c.376G>A	rs61758784	Exon 2	NS	A126T	Tolerated	0.005	No data	[5], [7], [8], [11]
c.404+72ins9bp	–	Intron 2				0.002		
c.458G>A	–	Exon 3	NS	G153D	Damaging	0.002		
c.564G>T	–	Exon 3	NS	K188N	Tolerated	0.002		
c.572-17G>T	–	Intron 3						[7]
c.640C>T	–	Exon 4	NS	R214C	Tolerated	0.002		
c.706-18T>C	rs56401264	Intron 4				0.002	No data	[9]
c.859A>G	rs28363317	Exon 6	NS	T287A	Damaging	0.016	0.017	[5], [7], [8], [10], [11], [12]
c.871G>A	–	Exon 6	NS	D291N	Tolerated	0.002		
c.904+34T>C	–	Intron 9						[7], [9], [10], [12]
c.1062A>G	–	Exon 9	S	A354A		0.005		

*synonomous (S) or non-synonomous (NS) amino acid change.

MAF = minor allele frequency.

### DNA Sequencing of *RAD51C*

The nine primer pairs used to amplify the 9 exons and intron-exon boundaries of *RAD51C* were described in Meindl et al [5]. Amplicons were sequenced in both directions using the BigDye terminator 3.1 cycle sequencing kit with sequencing performed on an 3130 Sequencer from Applied Biosystems Inc. (ABI). Sequencing traces were analyzed using SeqScape 2.5 (ABI) and by manual inspection.

### Prediction of functional mutations

The amino-acid substitution prediction programs, SIFT (http://sift.jcvi.org/) and Polyphen (http://genetics.bwh.harvard.edu/pph/), were used to predict the possible impact of non-synonymous variants. The algorithms in both programs use evolutionary conservation across species, as well as reference sequence alignments, physiochemical differences and the proximity of the substitution to predicted functional domains and/or structural features.

#### Plasmid construction

Yeast two-hybrid plasmid constructs for full-length human *RAD51B*, *RAD51C* and *XRCC3* cDNAs were the kind gift of Dr. David Schild (Lawrence Berkeley National Laboratory; Berkeley, CA). The *RAD51B* and *XRCC3* cDNAs were cloned into the Gal4 DNA-binding domain vector pGBT9, and the *RAD51C* cDNA was cloned into the transcriptional activation domain vector pGAD424 both of which were the kind gift of Dr. Stan Fields (HHMI University of Washington; Seattle, WA). The Phusion Site-directed Mutagenesis Kit (Finnzymes/New England Biolabs) was used for mutagenesis, and each mutated construct was sequenced across the entire insert to confirm the mutation.

#### Yeast two-hybrid analysis

The Matchmaker yeast two-hybrid kit was used according to the manufacturer's instructions (Clontech). The yeast strain Y187 was co-transformed with either the human *XRCC3* or *RAD51B* cDNAs cloned into pGBT9 and the *RAD51C* cDNA into pGAD424 and plated on synthetic medium lacking leucine and tryptophan. To quantitate the interactions between any two protein combinations, liquid β-galactosidase assays were performed on yeast colonies containing both pGBT9 and pGAD424 derivatives using *O*-nitrophenol-β-d-galactopyranoside (ONPG) as a substrate as outlined in the yeast protocols handbook (Clontech). Briefly, for each construct, single doubly transformed yeast colonies were inoculated into five ml of synthetic medium lacking leucine and tryptophan and grown overnight at 30°C. One ml of the overnight yeast culture was transferred to a culture tube containing four ml of medium containing yeast extract, peptone, dextrose, and adenine (YPDA) and grown with shaking for five h at 30°C. Culture densities were assessed by OD_600_. Three 1.5 ml aliquots of each sample (triplicates) were centrifuged for two min at 10,000 *g* and washed with one ml of Z buffer (16.1 g/l NaHPO_4_·7H_2_O, 5.5 g/l NaHPO_4_·H_2_O, 0.75 g/l KCl, 0.246 g/l MgSO_4_·7H_2_O). Cells were then resuspended in 100 µl of Z buffer and were subjected to three freeze–thaw cycles. To each tube, 700 µl of Z buffer plus β-mercaptoethanol and 160 µl of 4 mg/ml ONPG was added and the tube was incubated at 30°C until a yellow color developed. The reaction was stopped by adding 400 µl of 1 M NaCO_3_ and the samples were centrifuged at 14,000 rpm for 10 min. Product formation was assessed by determining OD_420_ at appropriate intervals (16 h for the XRCC3+RAD51C assays and 30 min for the RAD51B+RAD51C assays) and units of β-galactosidase (per ?) were calculated. The results from each combination represent the average of five to seven separate co-transformants assayed in triplicate, with standard error of the mean. P values were determined using the student T-test with Microsoft Office Excel software.

#### Immunoblot analysis

Fifty ml cultures of yeast containing the plasmids of interest were grown to mid log phase (OD_600_ = 0.6 to 0.7) in synthetic medium lacking leucine and tryptophan. The cells were collected by centrifugation at 4°C for for min at 5,000 rpm in a Sorvall SA600 rotor, washed twice in ice-cold PBS, and resuspended in 600 ul lysis buffer (50 mM HEPES, pH 7.5, 140 mM NaCl, 1% Triton-X100, 0.1% sodium deoxycholate, 1 mM EDTA, 1× Complete Protease Inhibitor Cocktail (Roche), and 10 mM PMSF). An equal volume of glass beads was added and each sample was vortexed five times for 30 sec followed by centrifugation at 4°C to remove the cell debris. The protein concentrations were determined using the Bradford protein assay (Pierce) and the lysates were stored at −80 °C. Prior to electrophoresis, an appropriate volume of cell lysate was diluted in 5× SDS sample loading buffer (250 mM Tris-HCl pH 6.8, 50% Glycerol, 4% SDS, 250 mM DTT, 0.1% Bromophenol Blue) and boiled for 5 min. Total cellular protein (15 µg) was fractionated by SDS/PAGE on NuPAGE 4–12% gradient gels (Invitrogen) and electrotransferred onto PVDF membranes. The membranes were probed with antibodies directed against the Gal4 activation domain (Clontech) or β-actin (mAbcam 8224, Abcam). Following incubation with primary antibody, detection was carried out using horseradish peroxidase-conjugated goat anti-mouse secondary antibody (Thermo Scientific). Membranes were incubated with Supersignal West Fempto substrate (Pierce), followed by exposure with BioMax XAR film (Kodak). Quantification of immunoblot signals was performed by densitometry using the GS-800 Calibrated Densitometer (Bio-Rad Laboratories).
